# Low‐birthweight infants born to short‐stature mothers are at additional risk of stunting and poor growth velocity: Evidence from secondary data analyses

**DOI:** 10.1111/mcn.12504

**Published:** 2017-08-25

**Authors:** Bireshwar Sinha, Sunita Taneja, Ranadip Chowdhury, Sarmila Mazumder, Temsunaro Rongsen‐Chandola, Ravi Prakash Upadhyay, Jose Martines, Nita Bhandari, Maharaj Kishan Bhan

**Affiliations:** ^1^ Centre for Health Research and Development Society for Applied Studies New Delhi India; ^2^ Independent Consultant 181, Rue Du Parc Jean Monnet 01630 St Genis Pouilly France; ^3^ Indian Institute of Technology New Delhi India; ^4^ Knowledge Integration and Translational Platform (KnIT) Biotechnology Industry Research Assistance Council (BIRAC) New Delhi India

**Keywords:** linear growth, low birthweight, maternal stature, short mother, stunting

## Abstract

Low‐birthweight (LBW) infants are at an increased risk of stunting and poor linear growth. The risk might be additionally higher in these infants when born to short mothers. However, this hypothesis has been less explored. The objective of this secondary data analysis was to determine the risk of linear growth faltering and difference in linear growth velocity in LBW infants born to short mothers (<150 cm) compared to those born to mothers with height ≥150 cm during the first year of life. This analysis uses data from a community‐based randomized controlled trial of 2,052 hospital‐born term infants with birthweight ≤2,500g from urban low–middle socioeconomic neighbourhoods in Delhi, India. Data on maternal height and infant birth length were available from 1,858 (90.5%) of the infants. Infant anthropometry outcomes were measured at birth, 3, 6, 9, and 12 months of age. We found that infants born to short mothers had around twofold higher odds of stunting and lower attained length‐for‐age *Z* scores compared to infants of mothers with height ≥150 cm, at all ages of assessment. Linear growth velocity was significantly lower in infants of short mothers particularly in the first 6 months of life. We conclude that LBW infants born to short mothers are at a higher risk of stunting and have slower postnatal growth velocity resulting in lower attained length‐for‐age *Z* scores in infancy. Evidence‐based strategies need to be tested to optimize growth velocity in LBW infants especially those born to short mothers.

**List of abbreviations**
LAZ: length‐for‐age *Z*
LBW: low birthweight
*SD*: standard deviation
WHO: World Health Organization
WHZ: weight‐for‐height *Z*

## 1 INTRODUCTION

Stunting affects approximately 162 million under‐five children and is a major public health problem globally (de Onis & Branca, 2016; Victora et al., 2008; World Health Organization [WHO], 2014). The Rapid Survey of Children in 2013–14 showed that 38.5% of the under‐five children in India are stunted (Rapid Survey of Children, 2013–14). Impaired linear growth in the first 1,000 days of life is related to poor health outcomes, cognitive development, and educational performance later in life (de Onis & Branca, 2016; Victora et al., 2008). Given the importance of the problem, the World Health Assembly resolution endorsed a comprehensive implementation plan on maternal, infant, and young child nutrition, which targets 40% reduction in the number of stunted under‐five children, by 2025, globally (WHO, 2014). Early identification of high‐risk population and targeted evidence‐based interventions to improve linear growth may help in achieving these goals.

Low‐birthweight (LBW) infants are at an increased risk of linear growth faltering (Christian et al., 2013; Prendergast & Humphrey, 2014). In India, 18.6% of the newborns are LBWs (Rapid Survey of Children, 2013–14). Recent data show that 41% of the childhood stunting in India is attributed to the risk factor cluster that includes low birthweight—intrauterine growth restriction and preterm (Danaei et al., 2016). This risk of stunting in the LBW infants may be additionally higher when they are born to short‐stature mothers; however, data supporting this hypothesis particularly in low‐ and middle‐income country settings are limited.

Evidence suggests that maternal short stature (<150 cm) predicts growth failure in children (Subramanian, Ackerson, Davey, & John, 2009) and are more likely to have a stunted child at 2 years (Addo et al., 2013). The interrelation between maternal stature and linear growth of the child is largely due to the shared genetic background and environmental determinants that affect the mother during her early childhood and development (Hernandez‐Diaz et al., 1999; Hirschhorn et al., 2001). This subsequently leads to a cycle of malnutrition and poor growth that follows across generations and affects growth of the offspring (Martorell & Zongrone, 2012). In short‐statured women, other physical mechanisms such as suboptimal development of pregnancy‐related anatomical systems and metabolic mechanisms such as maternal circulating glucose levels and reduced protein and energy stores may also contribute towards intrauterine growth restriction and subsequently postnatal poor linear growth in their infants (Hernandez‐Diaz et al., 1999). A better understanding of the growth trajectories, specifically the timing of growth faltering in LBW infants by maternal height would assist in the identification of a specific subgroup of an already vulnerable section of infants that should possibly be prioritized to receive additional linear growth‐promoting interventions.

In the current analyses, our objectives were to determine (a) the risk of linear growth faltering and (b) linear growth velocity in LBW infants born to short mothers (<150 cm) compared to mothers with height ≥150 cm in the first year of life, primarily at 12 months of age. Linear growth faltering was expressed as the risk of stunting and difference in attained length‐for‐age *Z* (LAZ) scores. As a secondary objective, we also explored the risk of infant stunting according to different subcategories of maternal stature, that is, less than 145, 145 to 149.9, 150 to 154.9, and ≥155 cm.

**Key messages**

- Low‐birthweight (LBW) infants born to short‐stature mothers have twofold higher risk of stunting during infancy and poor linear growth velocity in the first 6 months of life.
- Future research to examine the efficacy of integrated interventions starting from periconception and throughout pregnancy to achieve optimal linear growth in infants of short‐stature mothers may be of great value.
- Considering that 40% of the stunting in India is attributed to the LBW cluster, newer strategies to provide special support to these infants at least up to 6 months of life, especially when the mother is of short stature, may be beneficial.

## 2 METHODS

### 2.1 Design

In this secondary analyses, data from a community‐based, double‐blind, randomized, placebo‐controlled trial (Taneja et al., 2009) of 2,052 hospital‐born term infants with birthweight ≤2,500 g were analysed. The primary objective of the trial was to examine the effect of daily zinc supplementation on infant morbidity and growth (Clinicalhttp://trials.gov NCT00272142). The study participants were from urban neighbourhoods in New Delhi, India, and largely belonged to low and middle socioeconomic status. The study infants were randomized to receive either elemental zinc (*n* = 1,026) or placebo (*n* = 1,026). Data were collected between January 2005 and August 2007.

### 2.2 Procedures

Two tertiary care government hospitals, Hindu Rao and Kasturba Hospital, Delhi, were identified for enrolment of the study participants. Study workers visited the hospitals daily to identify new births. Families of newborns from the surrounding areas (within ≤10 km of the hospitals) were informed about the study and offered participation. If they were willing to participate, birthweight and length of the infants were measured by the study workers. LBW (≤2,500 g) infants, born at term, that is, >37 weeks gestational age (as per hospital records), were enrolled in the study. Written informed consent was obtained from the caregivers of the enrolled infants. Baseline sociodemographic information was collected at enrolment. Infants were home visited at ages 3, 6, 9, and 12 months, and anthropometry outcomes were measured at each of these visits.

All weight and length measurements were performed by trained research assistants of the study outcome measurement team. Standardization exercises for interobserver and intraobserver variability in weights and lengths were conducted. Portable weighing scale (Seca, Salter Scales, Germany) and length measurement boards (locally manufactured) measuring to the nearest 100 g and 0.1 cm, respectively, were used. The accuracy of weighing scales and measurement boards was checked daily against standard weights and standard steel rods, respectively. Standard procedures were followed to measure maternal height, to the nearest 0.1 cm, using Harpenden portable stadiometer. Details of the procedures have been described earlier (Taneja et al., 2009).

### 2.3 Study population and sample size

**Figure 1:**
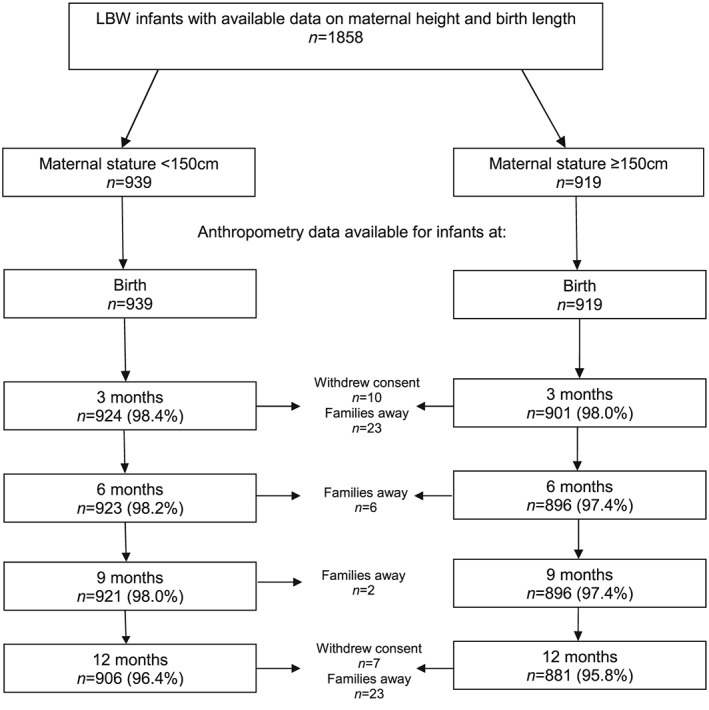
Available sample size at different time points of assessment. LBW = low birthweight

In the cohort of 2,052 hospital‐born term LBW mother–infant dyad that were enrolled in the main trial, data on maternal height and infant birth length were available for 1,858 (90.5%) participants. Of these, more than 95% infants (*n* = 1,787) were followed up till 1 year of age, and anthropometry measurements were done. The primary reason for loss to follow‐up was that families were away or withdrawal of consent for further participation (Figure 1).

With the available numbers (*n* = 1,787) of LBW infants from this cohort; we had >90% power to detect 10% difference in risk of stunting; 0.2 standard deviation (*SD*) difference in LAZ scores and 0.15 *SD* (i.e., 0.25 cm) difference in linear growth velocity at 12 months of age between LBW infants born to short‐stature mothers compared to those born to mothers with height ≥150 cm, at 5% alpha error. However, given the correlated nature of data due to multiple measurements in a single child, the power to compare linear growth outcomes across the two groups at intermediate time points ranged between 80% and 85%, considering an intraclass correlation coefficient of .6 (calculated from existing data) and 5% alpha error.

### 2.4 Exposure, outcomes, and covariates

#### 2.4.1 Exposure

Maternal short stature was defined as height <150 cm (−2 *SD* for a girl aged 19 years as per 2006 WHO growth standards; Addo et al., 2013; WHO, 2006). Mothers with height ≥150 cm were considered as the reference group. For subgroup analysis, maternal stature was subclassified into less than 145, 145 to 149.9, 150 to 154.9, and ≥155 cm (Kozuki et al., 2015). In these analyses, mothers with height ≥155 cm were considered as the reference category.

#### 2.4.2 Outcomes

Stunting was defined as LAZ score less than −2 *SD* (WHO, 2006). Linear growth velocity was defined as rate of change in length over the previous 3‐month period (WHO, 2009). Attained LAZ scores were calculated using WHO standards (WHO, 2006). Differences in attained mean LAZ scores in LBW infants, by maternal height, were calculated at birth, 3, 6, 9, and 12 months of age.

Additionally, as an exploratory exercise, we looked into the variation of weight‐for‐height *Z* (WHZ) scores by maternal height at birth, 3, 6, 9, and 12 months of age. WHZ scores were calculated using WHO (2006) standards.

#### 2.4.3 Covariates

Variables known to be associated with childhood stunting, through available literature, were considered as covariates (Rahman, Howlader, Masud, & Rahman, 2016; Stewart, Iannotti, Dewey, Michaelsen, & Onyango, 2013). Sociodemographic characteristics of the mother (age, years of schooling, occupation, monthly family income, and religion); maternal weight at enrolment; pregnancy‐related factors (birth spacing and type of delivery); infant factors (sex, birthweight, birth order, breastfeeding at enrolment, and number of hospitalizations in the previous 3 months from the time of assessment); and the intervention received; that is, zinc supplement were considered as potential covariates and were included in the regression models for adjustment.

### 2.5 Analyses

Analyses were done using STATA14.0 (Stata Corp., College Station, TX, USA). Proportions and means (*SD*) or median (interquartile range) were calculated for categorical and continuous variables by maternal stature, that is, <150 and ≥150 cm. Proportion of infants stunted, mean linear growth velocities, and mean LAZ scores at 3, 6, 9, and 12 months were calculated by maternal stature. All potential covariates were included in the respective multivariable models to adjust for confounding. Multivariable logistic regression analysis was done to examine the effect of maternal stature on stunting at different time points of measurement. Subgroup analysis was done to examine risk of stunting in LBW infants according to different categories of maternal height, considering height ≥155 cm as the reference category. Multivariable linear regression was done to explore effect of maternal stature on linear growth velocity for each three monthly time intervals. Interaction between maternal stature and all potential covariates were examined by including interaction terms in each regression model.

To estimate the effect of maternal short stature on attained infant LAZ scores, a multivariable linear mixed‐effect regression model with an unstructured covariance matrix (Johnson, Balakrishna, & Griffiths, 2013) was used. In this model, in order to account for the interdependence of multiple observation periods in the same child, time (age in months) of assessment was taken to be the level‐1 source of variation, with individual children at level 2. All potential covariates were included as fixed effect variables in this model. The interaction between maternal stature and time (age in months) of assessment on LAZ scores was found significant. Therefore, the interaction term was included in the model to obtain the independent effect of maternal stature at different ages. Moreover, to obtain individual effect sizes at each time of assessment including at birth, “contrast” command was used. Two different adjusted models were developed. In Model 1 all potential confounders were included as fixed effect variables, and in Model 2, birth length was included in addition to the factors included in Model 1. The objective of Model 2 was to examine the birth length‐adjusted difference in the LAZ scores between the groups that persisted at 3, 6, 9, and 12 months.

### 2.6 Ethical considerations

For the main trial, clearances were obtained from the ethics committee of All India Institute of Medical Sciences, the WHO Ethics Review Committee, and the Society for Applied Studies, Delhi. Written informed consent was obtained from all participants. To use data of primary trial for this secondary analysis, permission was taken from the study investigators.

## 3 RESULTS

In short‐stature mothers with height <150 cm (*n* = 939), the mean (±*SD*) height was 144.7 ± 3.4 as opposed to 153.9 ± 3.3 in those with height ≥150 cm (*n* = 919). Baseline characteristics of the two groups and infant anthropometry at different time points of measurement are summarized in Table 1. Around a fourth (26%) of the mothers with short height and one fifth (20%) with height ≥150 cm had never been to school. The median length of LBW infants of short mothers was lower compared to infants of mothers with height ≥150 cm at all time points of assessment. The proportion of stunted infants was higher among short mothers at all ages.

**Table 1:** Baseline characteristics and infant anthropometry by maternal stature^a^

	Maternal stature <150 cm (*n* = 939)	Maternal stature ≥150 cm (*n* = 919)
Sociodemographic factors		
Maternal age: mean (*SD*)	25.50 (3.9)	25.44 (3.9)
Maternal years of schooling: median (IQR)	6 (0; 9)	8 (4; 10)
Maternal occupation		
Not working/housewife	886 (94.3)	879 (95.6)
Working	53 (5.6)	40 (4.3)
Family income per month in INR: median (IQR)	4,000 (2,500; 6,000)	4,000 (3,000; 6,000)
Type of family: nuclear	354 (37.7)	312 (33.9)
Religion		
Hindu	643 (68.4)	548 (59.6)
Muslim	292 (31.1)	365 (39.7)
Others	4 (0.4)	6 (0.6)
Maternal weight at enrolment (kg): mean (*SD*)	44.5 (8.9)	49.9 (9.8)
Maternal height (cm): mean (*SD*)	144.7 (3.4)	153.9 (3.3)
Pregnancy‐related factors		
Birth spacing for the current child		
Primi	317 (33.7)	357 (38.8)
<24 months	162 (17.2)	160 (17.4)
24–36 months	210 (22.3)	171 (18.6)
>36 months	250 (26.6)	231 (25.1)
Type of delivery		
Caesarean	136 (14.4)	78 (8.4)
Vaginal delivery	803 (85.5)	841 (91.5)
Infant factors		
Sex: female	502 (53.4)	537 (58.4)
Birthweight (g): mean (*SD*)	2.32 (0.1)	2.34 (0.1)
Birth order		
1st	317 (33.7)	357 (38.8)
2nd	227 (24.1)	229 (24.9)
3rd	174 (18.5)	161 (17.5)
>3	221 (23.5)	172 (18.7)
Breastfeeding (at enrolment)		
Not breast fed	4 (0.4)	5 (0.5)
Exclusive	499 (53.1)	476 (51.8)
Predominant	279 (29.7)	288 (31.3)
Partial	157 (16.7)	150 (16.3)
Intervention (zinc) received	464 (49.4)	464 (50.4)
Infant anthropometry		
Infant length (cm): median (IQR)		
0 month	46.0 (45.2; 46.8)	46.3 (45.4; 47.1)
3 months	56.4 (55.1; 57.6)	57.1 (55.7; 58.3)
6 months	62.4 (61.1; 63.8)	63.4 (61.8; 64.8)
9 months	66.2 (64.5; 67.9)	67.2 (65.5; 68.9)
12 months	69.1 (67.3; 70.7)	70.3 (68.5; 72.1)
Infant stunting rates^b^		
0 month	404 (43.0)	297 (32.3)
3 months	484 (52.3)	290 (32.1)
6 months	429 (46.4)	245 (27.3)
9 months	491 (53.3)	305 (34.0)
12 months	559 (61.7)	361 (40.9)

*Note*. IQR = interquartile range; *SD* = standard deviation.

a The figures indicate numbers (%) unless indicated otherwise.

b *n* were different in the two groups at different time points. At 0 month 939/919; at 3 months 924/901; at 6 months 923/896; at 9 months 921/896; at 12 months 906/881.

In logistic regression analysis, LBW infants born to short‐stature mothers were found to have twofold higher odds of stunting compared to those born to mothers with height ≥150 cm, regardless of the age of assessment, that is, at birth, 3, 6, 9, and 12 months of age. In the subgroup analysis with different maternal height categories, the risk of infant stunting was highest in mothers who were <145 cm compared to mothers with height ≥155 cm. This finding was consistent across all ages of assessment (Table 2). A dose response gradient was observed showing that the shorter the mothers, the higher the risk of infant stunting.

**Table 2:** Maternal stature and risk of stunting in low‐birthweight infants at different time points of measurement

Exposure maternal stature (cm)	*n*	Infant stunting at 3 months		*n*	Infant stunting at 6 months		*n*	Infant stunting at 9 months		*n*	Infant stunting at 12 months	
		Unadjusted OR [95% CI]	Adjusted^a^ OR [95% CI]		Unadjusted OR [95% CI]	Adjusted^a^ OR [95% CI]		Unadjusted OR [95% CI]	Adjusted^a^ OR [95% CI]		Unadjusted OR [95% CI]	Adjusted^a^ OR [95% CI]
≥150	901	1.00	1.00	896	1.00	1.00	896	1.00	1.00	881	1.00	1.00
<150	924	2.31 [1.91, 2.80]	2.18 [1.76, 2.71]	923	2.30 [1.89, 2.80]	2.20 [1.76, 2.73]	921	2.21 [1.83, 2.67]	1.98 [1.60, 2.44]	906	2.32 [1.91, 2.80]	2.09 [1.69, 2.58]
Subgroups												
≥155	336	1.00	1.00	336	1.00	1.00	336	1.00	1.00	332	1.00	1.00
150–154.9	565	1.80 [1.33, 2.44]	1.67 [1.20, 2.30]	560	1.72 [1.25, 2.37]	1.62 [1.15, 2.26]	560	1.53 [1.14, 2.05]	1.40 [1.03, 1.91]	549	1.41 [1.06, 1.86]	1.28 [0.95, 1.72]
145–149.9	553	2.95 [2.19, 3.98]	2.81 [2.03, 3.90]	552	2.58 [1.88, 3.53]	2.48 [1.77, 3.47]	550	2.29 [1.71, 3.07]	2.09 [1.53, 2.84]	538	2.23 [1.68, 2.96]	2.03 [1.50, 2.74]
<145	371	4.22 [3.05, 5.83]	3.62 [2.51, 5.23]	371	4.71 [3.37, 6.58]	4.37 [3.01, 6.34]	371	4.14 [3.02, 5.68]	3.38 [2.38, 4.79]	368	4.30 [3.13, 5.91]	3.56 [2.50, 5.05]

*Note*. OR = odds ratio; CI = confidence interval; *SD* = standard deviation.

Adjusted for variables maternal age, maternal occupation, maternal years of schooling, family income, religion, maternal weight, birth order, birth spacing for current pregnancy, type of delivery, infant sex, birthweight, number of hospitalizations in each three monthly periods, and intervention (zinc supplement).

Linear regression analysis showed that the difference in linear growth velocity was highest in the birth to 3‐month period where babies of short mothers gained length poorly. This difference remained statistically significant (*p* < .05) up to 6 months of age. During the 6‐ to 9‐month and 9‐ to 12‐month periods, there were no statistically significant differences in growth velocities between the two groups (Table 3).

**Table 3:** Linear growth velocities in low‐birthweight infants of short mothers and mothers with height ≥150 cm

Time window (months)	Linear growth velocity in infants: rate of change of length (cm) per 3‐month mean (*SD*)		Difference in growth velocity in infants of short mothers compared to mothers with height ≥150 cm	
	Maternal stature <150 cm	Maternal stature ≥150 cm	Unadjusted coefficient [95% CI]	Adjusted^a^ coefficient [95% CI]
0–3	10.35 (1.67)	10.78 (1.67)	−.42 [−.57, −.27]*	−.39 [−.55, −.23]*
3–6	6.02 (1.53)	6.30 (1.49)	−.27 [−.41, −.13]*	−.26 [−.41, −.12]*
6–9	3.74 (1.18)	3.87 (1.43)	−.13 [−.25, −.01]*	−.07 [−.20, .05]
9–12	2.96 (1.05)	3.03 (1.26)	−.07 [−.18, .03]	−.04 [−.15, .07]

*Note*. CI = confidence interval; *SD* = standard deviation.

a Results from multivariable linear regression model, with infants of mothers with height ≥150 cm as reference group. Adjusted for variables maternal age, maternal occupation, maternal years of schooling, family income, religion, maternal weight, birth order, birth spacing for current pregnancy, type of delivery, infant sex, birthweight, number of hospitalizations in each three monthly periods, and intervention (zinc supplement).

* Significant difference between groups (*p* < .05).

**Figure 2:**
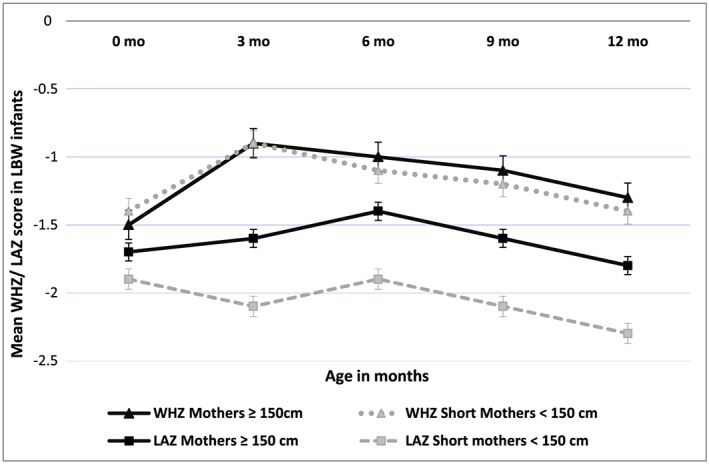
Three monthly mean weight‐for‐height *Z* (WHZ) and length‐for‐age *Z* (LAZ) scores in low‐birthweight (LBW) infants of short mothers and mothers with height ≥150 cm

Figure 2 shows that the trajectories of attained LAZ mean scores among the two groups were clearly different (*p* < .01), whereas trajectories for attained WHZ mean scores were similar throughout infancy and remained above −2 *SD*. In infants of short‐stature mothers, the LAZ scores continued to decline after birth and went below −2 *SD* at 3 months. In infants of mothers with height ≥150 cm, scores improved after birth until 6 months and consistently remained above −2 *SD*. After 6 months of age, LAZ scores declined in both groups.

Results from linear mixed‐effects model showed that LAZ scores in infants of short‐stature mothers were significantly lower than infants of mothers with height ≥150 cm (*p* < .001) at all time points of measurement (Model 1, Table 4). In Model 2, after additional adjustment for birth length, the LAZ scores in infants of short mothers remained significantly lower than those in infants of mothers with height ≥150 cm (*p* < .001) at all time points (Table 4).

**Table 4:** Attained LAZ scores in low‐birthweight infants of short mothers and mothers with height ≥150 cm

Age of the child (months)	Infant attained LAZ scores mean (*SD*)		Difference in attained LAZ scores in infants of short mothers compared to mothers with height ≥150 cm		
	Maternal stature <150 cm	Maternal stature ≥150 cm	Unadjusted coefficient [95% CI]	Model 1	Model 2
				Adjusted^a^ coefficient [95% CI]	Adjusted^b^ coefficient [95% CI]
0	−1.90 (0.68)	−1.72 (0.69)	−.18 [−.27, −.10]	−.10 [−.18, −.02]	−
3	−2.05 (0.97)	−1.66 (0.98)	−.39 [−.49, −.32]	−.32 [−.40, −.24]	−.21 [−.28, −.14]
6	−1.96 (0.99)	−1.45 (1.02)	−.51 [−.61, −.43]	−.43 [−.51, −.35]	−.32 [−.40, −.25]
9	−2.12 (1.02)	−1.57 (1.10)	−.55 [−.64, −.46]	−.46 [−.54, −.37]	−.35 [−.43, −.28]
12	−2.34 (1.01)	−1.80 (1.07)	−.54 [−.63, −.46]	−.46 [−.54, −.38]	−.35 [−.42, −.28]

*Note*. LAZ = length‐for‐age *Z*; CI = confidence interval; *SD* = standard deviation.

a Results from linear mixed‐effects model. Infants of mothers with height ≥150 cm are the reference group. Adjusted for variables maternal age, maternal occupation, maternal years of schooling, family income, religion, maternal weight, birth order, birth spacing for current pregnancy, type of delivery, infant sex, birthweight, number of hospitalizations in each three monthly periods, and intervention (zinc supplement).

b This model is adjusted for all factors mentioned above and length at birth.

## 4 DISCUSSION

Our study showed that LBW infants born to mothers with height ≤150 cm had a comparatively higher risk of stunting with lower attained LAZ scores in their infancy, compared to those born to mothers with height ≥150 cm, after adjusting for all potential confounding factors. Statistically significant differences in linear growth velocity among the two groups were observed in the first 6 months of life, a period when the growth of infants of short mothers was slower. The difference in growth velocity between the two groups reduced with increasing age of the infant and failed to achieve statistical significance after 6 months of age.

LBW is one of the major risk factors associated with infant stunting (Danaei et al., 2016; Prendergast & Humphrey, 2014). However, data on the additional risk of stunting in this already vulnerable group of LBW infants when born to short mothers are limited. Our analysis showed that LBW infants born to short mothers had twofold higher odds of stunting during infancy; this supports the intergenerational nature of stunting in LBW infants.

Lower growth velocity in the initial months and comparatively lower LAZ scores in LBW infants of short mothers as observed in our study are in agreement with documented findings in animal models (Wu, Bazer, Cudd, Meininger, & Spencer, 2004). It might possibly reflect that growth retardation actually begins from foetal life due to inadequate nutrient transfer across the placenta because of poor nutritional status in short‐stature mothers and constrained intrauterine environment (Baptiste‐Roberts et al., 2009; Marsal, 2002; Toh‐Adam, Srisupundit, & Tongsong, 2012; Wu et al., 2004). The nutritional insult during critical periods of intrauterine growth may lead to a phenomenon known as the “foetal programming effect” (Fall, 2003; Wu et al., 2004). Interestingly, in animal models, higher weight gain has been observed at the cost of muscle or skeletal growth when piglets were placed on a normal diet after being exposed to undernutrition in foetal or early postnatal life (Fall, 2003; McCance, 1962). This seems to be in concurrence with our study findings where we observed similar WHZ scores in LBW infants of the two groups but significantly lower LAZ scores in infants of short‐stature mothers. This suggests higher risk of stunting in these infants but no additional risk of wasting.

Previous estimates (Victora, de Onis, Hallal, Blossner, & Shrimpton, 2010) show that there is a 0.7‐unit deficit in LAZ score at birth in Indian children that declines further to reach −1.4 at 12 months of age. In the current study population of LBW infants, the change in the trajectories of the LAZ scores over time was clearly differential by maternal stature. The mean LAZ scores at birth were −1.7 and −1.9 in children born to mothers with height ≥150 and <150 cm, respectively. At 12 months, the mean LAZ scores in the LBW infants of the former group remained at −1.8, whereas that in the latter group declined further to −2.3. The “fork‐like” appearance of the LAZ scores in the initial 3 months after birth between the two groups as seen in Figure 2 is noteworthy. During this time, the LAZ scores in infants born to short‐stature mothers deteriorated and went below −2 *SD* whereas that in infants born to mothers with height ≥150 cm improved and remained well above −2 *SD*. Growth faltering during this time period is critical and may have long‐term health consequences (Danaei et al., 2016; Victora et al., 2008). Early nutritional and healthcare interventions in the short mothers from periconception or pregnancy and additional support to their LBW infants from birth may be helpful to narrow this gap in postnatal catch‐up growth and reduce infant stunting; however, this needs further research (Ramakrishnan, Grant, Goldenberg, Zongrone, & Martorell, 2012). In focusing our efforts to accelerate linear growth in these subset of infants, we should be cognizant of the fact that inadvertently we might introduce them to the risk of developing noncommunicable diseases. However, from what best we know currently, interventions to accelerate early catch‐up growth before 2 years of age is seldom associated with long‐term risk of developing noncommunicable diseases (Martin, Connelly, Bland, & Reilly, 2017; Victora et al., 2008). Even so, there is a need to be cautious as the evidence is sketchy for LBW infants (Martin et al., 2017).

Birth length is known to be an indicator of intrauterine growth (Hindmarsh, Geary, Rodeck, Kingdom, & Cole, 2002). We observed a difference of 0.1 LAZ scores between the two groups at birth. Moreover, when adjusted for birth length in addition to other confounding factors, the LAZ scores in the infants of short‐stature mothers remained significantly lower compared to infants of mothers ≥150 cm at all subsequent time points of measurement. This finding suggests that maternal short stature not only influences intrauterine growth restriction but also affects the postnatal linear growth of infants. Apart from genetic factors that explain only around 10% of the growth faltering (Lango Allen et al., 2010), the poor linear growth during postnatal life in children of short mothers may be explained by factors such as inadequate breast milk volume or poor nutritive quality of breast milk or poor lactation performance in these mothers who are chronically undernourished, as reported from animal models and some human studies (Allen, 1994; Chapman & Nommsen‐Rivers, 2012; Rasmussen, 1992). However, this needs further research.

Our analysis had several strengths and some limitations. First, we had high‐quality anthropometric data on LBW mother–infant dyads at five time points throughout infancy that was adequately powered to make valid comparisons between the groups on outcomes related to linear growth at 12 months of age and also at other intermediate time points of measurement (3, 6, and 9 months). Second, our data were limited to hospital‐born term LBW babies >37 weeks gestation. Due to unavailability of reliable data on gestational age, analyses of infant growth patterns based on the weeks of gestation could not be performed. Further research to compare the effect of maternal stature on growth trajectories of preterm, small‐for‐gestational age, and normal birthweight babies would be interesting. Third, the data on breastfeeding practices were limited. Data on breastfeeding practices at enrolment were included in the analyses, but those on later time points were not available. Published literature is suggestive of the fact that breastfeeding has a profound effect on survival and infection prevention in the first 6 months of life (Sankar et al., 2015) but has somewhat a limited role on child linear growth (Giugliani, Horta, Loret de Mola, Lisboa, & Victora, 2015). On the basis of this proposition, we might argue that noninclusion of breastfeeding status in the model will not alter the current findings to an extent that the nature of the argument will change radically. However, we do realize that although breastfeeding might not have a direct effect on linear growth, the protective effect may help avoid stunting through the infant experiencing less infection. Last, we also acknowledge the limitation of the observed associations in these secondary data analyses that may be due to unavailability of information on other plausible factors related to infant stunting such as paternal height, mother's dietary intake, water, sanitation and hygiene practices, and complementary feeding practices. Future primary data studies to examine epidemiological associations between maternal stature and linear growth of LBW infants in greater detail may be useful.

## 5 CONCLUSIONS

Our analysis suggests maternal stature to be an independent predictor of stunting in LBW infants. Initial lag in linear growth velocity in the first 6 months of postnatal life in LBW infants born to short‐stature mothers culminates into lower attained LAZ scores compared to the infants of mothers with height ≥150 cm. This pushes the argument for special efforts on two fronts: first, special care of LBW infants at least up to 6 months of life to maximize linear growth with greater emphasis when the mother is short statured and, second, strategies to promote optimal foetal growth combined with others to accelerate early postnatal growth velocity among LBW infants. In this regard, providing evidence‐based interventions starting from periconception till infancy are important (Martorell & Zongrone, 2012; Ramakrishnan et al., 2012). Recognizing that nutritional interventions during periconception or pregnancy are likely to increase the size of the baby, adequate delivery care must be ensured to address the concern of any potential obstructed labour in short‐stature mothers (Konje & Ladipo, 2000). Literature suggests that though height is a heritable trait, only around 10% is explained by genetic factors and the major contribution is by environmental factors that may be modifiable (Lango Allen et al., 2010). Further research to examine whether optimal linear growth can be attained in infants of short mothers with integrated interventions starting from periconception, throughout pregnancy and infancy, without increasing the risk of obesity may be of immense value.

## CONFLICTS OF INTEREST

The authors declare that they have no conflicts of interest.

## CONTRIBUTIONS

BS was involved in conceptualizing research questions; preparation of data file; statistical analysis; data interpretation; and manuscript writing, editing, and finalization. ST was involved in the preparation of data file, data interpretation, and manuscript review. RC was involved in statistical analysis and manuscript review. SM, TRC, RPU, JM, NB, and MKB were involved in revising the manuscript critically for important intellectual content. All authors have read and approved the final manuscript.
